# Gender Identity Milestones, Minority Stress and Mental Health in Three Generational Cohorts of Italian Binary and Nonbinary Transgender People

**DOI:** 10.3390/ijerph18179057

**Published:** 2021-08-27

**Authors:** Cristiano Scandurra, Agostino Carbone, Roberto Baiocco, Selene Mezzalira, Nelson Mauro Maldonato, Vincenzo Bochicchio

**Affiliations:** 1 Department of Neuroscience, Reproductive Sciences and Dentistry, University of Naples Federico II, 80131 Naples, Italy; nelsonmauro.maldonato@unina.it; 2 Department of Developmental and Social Psychology, Sapienza University of Rome, 00185 Rome, Italy; agostino.carbone@uniroma1.it (A.C.); roberto.baiocco@uniroma1.it (R.B.); 3 Department of Philosophy, Sociology, Education and Applied Psychology, University of Padova, 35139 Padova, Italy; selene.mezzalira@gmail.com; 4 Department of Humanistic Studies, University of Calabria, 87036 Cosenza, Italy; vincenzo.bochicchio@unical.it

**Keywords:** transgender, minority stress, mental health, generations, identity, milestones, binary, nonbinary, coming out, life course

## Abstract

Transgender and gender nonconforming (TGNC) people experience high rates of minority stress and associated risk for negative health outcomes. However, during the last years, significant positive socio-cultural changes have happened, and younger cohorts of TGNC individuals are having diverse experiences compared to older cohorts. By integrating the minority stress theory and the life course perspective, this cross-sectional, web-based study aimed to explore in 197 Italian TGNC people aged 18 to 54 years (*M* = 29.82, *SD* = 9.64) whether the average ages of gender identity milestones (i.e., first insights about being TGNC, self-labeling as a TGNC person, and coming out), minority stress, and mental health vary among three generational cohorts (i.e., Generation Z, Millennials, and Generation X). Compared with older cohorts, younger participants: (a) were more likely to be in the trans-masculine spectrum; (b) self-labeled as TGNC and came out earlier; (c) had more negative expectations and lower levels of disclosure; and (d) had higher levels of mental health problems. No generational differences related to first insights about being TGNC and distal minority stressors were found. Furthermore, compared with binary individuals, participants with a non-binary identity: (a) reported later ages for the gender identity milestones; (b) had higher negative expectations; and (c) had higher levels of mental health problems. Overall, our findings indicated that changes in the social environments have a limited impact on stigmatization processes and mental health of Italian TGNC people.

## 1. Introduction

The term transgender and gender nonconforming (TGNC) refers to people whose gender identity, expression, or behavior differ from those typically associated with their sex assigned at birth [1]. TGNC individuals may have a binary or nonbinary gender identity. Those with a binary gender identity tend to identify themselves as women if assigned male at birth (AMAB) or men if assigned female at birth (AFAB). On the contrary, TGNC individuals with a nonbinary gender identity tend to identify themselves with neither exclusively masculine nor feminine gender, rejecting a binary view of gender identity [2].

During the last decades, a growing body of research has elucidated the psychosocial processes underlying the impressive rates of adverse health outcomes reported by TGNC people [3,4]. Indeed, within the minority stress theory (MST) [5,6]—that is, the predominant theoretical framework in the field of health disparities of both sexual (i.e., lesbian, gay, and bisexual (LGB)) and gender (i.e., TGNC) minority groups—previous research highlighted that TGNC people are a strongly stigmatized population. Stigmatizing experiences increase the likelihood of developing negative mental health outcomes (e.g., depression, anxiety, and general distress) via the internalization of TGNC identity’s negative social evaluation [7,8]. In other words, TGNC health disparities would stem from the stigmatizing social environment, which leads TGNC individuals to experience a chronic, unique, and socially-based stress consisting of: (a) distal stressors (e.g., objective stressors, such as discrimination, victimization, rejection, or non-affirmation of gender identity) and (b) proximal stressors (e.g., subjective stressors, such as internalized transphobia, negative expectations for future events, or nondisclosure) [5]. Notwithstanding, a significant body of research has also highlighted that TGNC people represent a resilient population able to activate both individual- (e.g., identity pride) and community-level (e.g., community connectedness) resilience factors to protect themselves from the adverse effects of stigma on health [9,10,11,12].

TGNC people are becoming more and more visible in society, and many significant socio-cultural changes have happened in the last years. For instance, there has been a shift in the mental health community that has taken a slow, depathologizing path of TGNC identities. Indeed, the latest versions of the Diagnostic and Statistical Manual of Mental Disorders (DSM–5) [13] and the International Classification of Diseases (ICD-11) [14] declared that the incongruence between one’s perceived gender and the sex assigned at birth can no longer be considered a pathological condition. Similarly, a significant shift has been observed with respect to the perceived gender identity of TGNC individuals, with younger cohorts being more likely to self-identify within a nonbinary spectrum of gender compared with older cohorts of TGNC people [15,16,17]. These two examples could represent significant instances of detecting a socio-cultural change around attitudes and perceptions of TGNC identity, and it is plausible that TGNC individuals themselves are influenced by these changes in the ways they experience their own identity as well as in the rates of stigmatization and, therefore, of health problems. Indeed, the most recent research trend in the tradition of studies concerning the LGB and TGNC health consists in matching the MST with the life-course perspective (LCP)—that is, a useful framework to analyze people’s health needs and experiences over the course of their life—to detect potential differences in terms of life experiences, stress, and health, and to construct a culturally sensitive picture of this population [17,18,19].

While the MST addresses the relationships between social status, stress, and health, the LCP [20] analyzes how differences in the socio-cultural context may shape the identities and experiences of diverse generational cohorts. A generational cohort is characterized as individuals born during the same time range and having experienced similar social events in their life [21], building analogous collective memories [22]. Thus, examining generational cohorts allows assessing the roles of socio-cultural context and individual differences. As the premise of the MST is that minority stress should be understood in the social context, and during the last years, many positive changes occurred in the social environment for both sexual (i.e., LGB) and gender (i.e., TGNC) minority individuals, the few studies matching the MST and LCP started from the hypothesis that younger cohorts would experience lower levels of minority stress and, as a consequence, lower mental health problems than older cohorts [17,23,24,25]. Indeed, some research in the sociological field talked about a general decline of sexual prejudice in social contexts [26,27,28].

Furthermore, as the social contexts seem more and more accepting towards LGB and TGNC individuals, common milestones of sexual and gender identity in these individuals were hypothesized to occur earlier in younger cohorts than in older ones. For instance, gender identity milestones in TGNC individuals mainly concern: (a) the age of first insights about being TGNC; (b) the age at which they self-labeled as a TGNC person; (c) the time at which they came out as TGNC; and (d) the age at which they started to live full time as a TGNC person and underwent some kind of medical gender-affirmation procedure (e.g., hormonal treatment) [15]. However, the few research in this field—conducted with U.S. samples—reported that, although sexual and gender identity milestones varied significantly among cohorts, with younger participants reporting earlier ages for the milestones compared with older cohorts, neither minority stress nor mental health resulted improved in younger cohorts both in LGB [24] and TGNC [17] samples; on the contrary, mental health was worse in the younger cohorts than the older ones. These studies concluded that changes in the social contexts would have a limited impact on stress processes and, therefore, on the mental health of minority groups, highlighting the persistence of cultural, ideological systems that perpetuate differences in identity status (e.g., homophobia, transphobia, cisgenderism, and heteronormativity) and reproduce health disparities.

In Italy, that is the context of the current study, except for one study analyzing the coming out milestone in three generations of Italian sexual minority people [29], no previous studies have assessed gender identity milestones, minority stress, and mental health of TGNC individuals matching the MST and LCP by considering different generational cohorts. However, some studies have applied the MST to samples of Italian TGNC individuals, highlighting that the Italian socio-cultural context is not highly supportive for these people [30,31,32,33,34]. Indeed, although TGNC individuals are becoming very visible in Italy, participating in public talk shows or obtaining significant political roles, they continue to experience high levels of victimizations and struggle to obtain legal recognition that could improve their access to health resources [35]. For example, although a sentence delivered by the Italian Court of Cassation in 2015 allowed TGNC individuals to change their legal name without any gender-affirmation procedures, the official legislation dating back to 1982 has never been formally changed [36].

Thus, within the MST and the LCP, the current study aimed to explore whether the gender identity milestones, minority stress, and mental health vary among different generational cohorts of Italian TGNC individuals. Based on the studies by Meyer et al. [24] and Puckett et al. [17], we hypothesized that: (1) younger generations will report higher percentages of nonbinary identity than older counterparts; (2) younger cohorts will report earlier ages for the milestones compared with older cohorts; (3) levels of minority stressors will not vary among different generational cohorts; and (4) younger cohorts will report worse levels of mental health than older counterparts. Furthermore, we also explored the role of sex assigned at birth (AMAB vs. AFAB) and gender identification (binary vs. nonbinary), as both of these variables are crucial dimensions in minority stress experiences and mental health [17]. However, while previous studies seem to clearly report that nonbinary individuals are at higher risk of stigmatization and mental health problems than their counterparts [4], findings of studies assessing the impact of sex assigned at birth on minority stress and mental health are less robust. For instance, Bockting et al. [3] found that AMAB people experienced lesser stigmatizing experiences than AFAB counterparts, reporting higher levels of one only form of mental health problem (i.e., depression), whereas AFAB participants experienced higher levels of another form of mental health problem (i.e., somatization). Similarly, Scandurra et al. [12] found that, although AMAB participants reported higher levels of one form of minority stress (i.e., rejection) than AFAB counterparts, AFAB individuals experienced higher levels of another minority stressor (i.e., internalized transphobia) than AMAB participants. Thus, because of these mixed results, we explored whether sex assigned at birth was relevant to the outcome variables without a specific hypothesis.

## 2. Materials and Methods

### 2.1. Procedures

The data of the current study were collected through a cross-sectional, web-based survey (Qualtrics software) within the project “Stress and Resilience in Trans Population Survey”, an Italian study aimed at assessing minority stress and health in Italian TGNC people. Participants were reached through advertisements published on online groups (e.g., Facebook groups and pages) specifically addressed to TGNC issues. Additionally, participants were involved through a snowball recruitment procedure by asking people interested in the survey and Italian stakeholders in the TGNC community to share the survey with potential interested participants. Participants were directed to the first page of the survey by clicking on the link, where information about researchers, objectives, study design, time of completion, benefits, and risks were provided. Participants were also informed about the anonymity of the survey.

The Italian Observatory on Gender Identity financed the study. Funds were used to incentivize the participation and completion of the survey. Indeed, participants were told that the completion of the survey allowed them to enter into a lottery, with the possibility to be extracted and receiving 50 €. Participants who expressed the intention to participate in the lottery were asked to report their email address voluntarily. To guarantee anonymity, participants were informed that only the principal investigator had access to this information and was obligated to disaggregate from the dataset the email of participants who decided to participate in the lottery. At the end of the recruitment phase, emails of participants who accepted to take part in the lottery were extracted, and participants were contacted by the principal investigator, who asked them to provide their bank details.

The project was designed in respect of the principles of the Declaration of Helsinki on Ethical Principles for Medical Research Involving Human Subjects and was approved by the ethical committee of the University of Calabria (protocol number: 28058; date of approval: 26 October 2018).

### 2.2. Participants

The survey was launched online between November 2018 and April 2019. Inclusion criteria were: (1) being at least 18 years old (the Italian age of consent); (2) have been living in Italy for at least 10 years; (3) speaking Italian language; and (4) self-identifying as TGNC (transgender, gender nonconforming, nonbinary, etc.). A total of 203 participants completed the survey.

Generational cohort groups were determined based on the recent study by Puckett et al. [17]. Specifically, participants were classified as: (a) Generation Z if born between 1997 and 2012; (b) Millennials if born between 1981 and 1996; (c) Generation X if born between 1965 and 1980; and (d) Boomers if born before 1964. Age was subtracted from the year of completing the study. Thus, the final ranges were: (1) 18–22 years for Generation Z; (2) 23–38 years for Millennials; (3) 39–54 years for Generation X; and (4) 55+ years for Boomers. However, due to the very low number of participants categorized as Boomers (*n* = 6), they were removed from the final sample. Thus, we conducted analyses on 197 TGNC participants considering three generational cohorts (i.e., Generation Z, Millennials, and Generation X). Furthermore, due to the relatively low sample size and with the need of reducing categorizations, analyses were not performed considering all the specific gender identities of participants but rather classifying the sample into binary (i.e., women, men, transwomen, and transmen) vs. nonbinary (e.g., genderqueer, genderfluid) gender identity.

Individuals in the final sample had an average age of 29.82 years old (*SD* = 9.64, range = 18–54). Sixty-three (32%) participants were AMAB, 152 (77.2%) had a binary gender identity, 100 (50.8%) had an educational level ≤ high school, and 187 (94.9%) were Caucasian. Description of the sample characteristics for each generational cohort are reported in Table 1.

### 2.3. Measures

#### 2.3.1. Demographics

Sociodemographic variables included age, sex assigned at birth (male, female, or other with specification), gender identity (women, men, transwomen, transmen, genderqueer, cross-dresser, and other with specification), ethnicity (Caucasian vs. non-Caucasian), and level of education (≤high school vs. ≥college). Regarding gender identity, participants were categorized as binary if they self-identified as women, men, transwomen, or transmen and nonbinary if they self-identified as outside the binary categorization of gender (e.g., genderqueer, genderfluid, nonbinary).

#### 2.3.2. Gender Milestones

Based on the survey conducted by Testa et al. [37], we asked participants three questions about: (1) the age at which they had first insights about being TGNC (i.e., “At what age did you first feel your gender might be different from the sex you were assigned at birth?”); (2) the age at which they self-labeled as a TGNC person (“At what age did you first identify as trans, genderqueer, two-spirit, or any gender identity other than the sex you were assigned at birth?”); and (3) the age at which they came out as a TGNC person (“At what age did you first tell someone else you were a gender other than the sex assigned to you at birth?”). Participants were allowed to skip these questions, as not all milestones can apply to everyone. As the current study was not specifically constructed to analyze generational cohorts but rather minority stress and health, we did not ask questions about the age at which participants started to live full time as a TGNC person and underwent the first medical gender-affirmation procedure.

#### 2.3.3. Gender Minority Stress

Distal and proximal minority stress were measured through some subscales of the *Gender Minority Stress and Resilience Scale* (GMSR) [36,37]. Specifically, distal stressors were assessed through the following subscales: (1) *gender-related discrimination* (*α* = 0.60), which assesses by 5 items different forms of discrimination (e.g., “I have had difficulty getting medical or mental health treatment (transition-related or other) because of my gender identity or expression”); (2) *gender-related rejection* (*α* = 0.64), which assesses by 6 items different forms of rejection (e.g., “I have been rejected or distanced from friends because of my gender identity or expression”); (3) *gender-related victimization* (*α* = 0.76), which assesses by 7 items different forms of victimization (e.g., “I have been threatened with physical harm because of my gender identity or expression”); and (4) *non-affirmation of gender identity* (*α* = 0.92), whose 6 items reflect different experiences of gender identity non-affirmation (e.g., “I have to work hard for people to see my gender accurately”). Proximal stressors were assessed through the following subscales: (1) *internalized transphobia* (*α* = 0.90), which assesses by 8 items shame towards one’s own TGNC identity (e.g., “My gender identity or expression makes me feel like a freak”); (2) *negative expectations for future events* (*α* = 0.90), which assesses by 9 items negative expectations related to the expression of one’s own gender identity or history (e.g., “If I express my gender identity/history, most people would think less of me”); and (3) *nondisclosure* (*α* = 0.78), which assesses by 5 items different means of nondisclosure used by TGNC individuals (e.g., “Because I don’t want others to know my gender identity/history, I pay special attention to the way I dress or groom myself”).

Response options for gender-related discrimination, gender-related rejection, and gender-related victimization were “never”, “yes, before age 18”, “yes, after age 18”, and “yes, in the past year”. Participants could click all options that apply to them. Responses were coded as 0 if “never” and 1 if “yes” at any point and then summed to obtain a total score. Instead, response options for all other subscales were from “strongly disagree” to “strongly agree” on a 5-point Likert scale. The total score was obtained by dividing the raw total score by the number of items, thus ranging from 1 to 5.

#### 2.3.4. Anxiety

Anxiety was measured with the *Severity Measure for Generalized Anxiety Disorder—Adult* [38,39], a 10-item questionnaire assessing the severity of anxious symptoms over the last 7 days. The response options ranged from 0 (“never”) to 4 (“all the time”). An example item is “During the past 7 days, felt anxious, worried, or nervous about social situations.” The total score was obtained by dividing the raw total score by the number of items, thus ranging from 0 to 4, with higher scores reflecting greater anxious symptoms. The *α* coefficient for the current sample was 0.90.

#### 2.3.5. Depression

Depression was measured with the short version of the *DSM-5 Severity Measure for Depression–Adult* [39,40], a 9-item questionnaire assessing the severity of depressive symptoms over the last 7 days. The response options ranged from 0 (“not at all”) to 3 (“nearly every day”). An example item is “Over the last 7 days, how often have you been bothered by feeling down, depressed, or hopeless?” The total score was obtaining by summing the score provided to each answer, thus ranging from 0 to 27, with higher scores indicating greater depressive symptoms. The *α* coefficient for the current sample was 0.89.

#### 2.3.6. Psychological Distress

The 12-item version of the *General Health Questionnaire* [41,42] was used to assess psychological distress over the past few weeks. Items were rated on a 4-point scale, from 0 (“less than usual”) to 3 (“much more than usual”). An example item is “Been able to concentrate on whatever you are doing.” The total score ranged from 0 to 36, with higher scores reflecting a greater degree of psychological distress. The *α* coefficient for the current sample was 0.92.

### 2.4. Statistical Analyses

All statistical analyses were performed using SPSS version 26, setting the level of significance at 0.05. Missing data only concerned the questions on gender identity milestones. Specifically, 2% (*n* = 4) of the sample did not answer the first question, 0.5% (*n* = 1) did not answer the second question, and 1% (*n* = 2) did not answer the third question. Thus, due to this very low percentage, missing data were treated using list-wise deletion.

First, we performed analyses to provide descriptive information of the sample. In this context, we assessed relationships between demographics (i.e., sex assigned at birth, gender identity, ethnicity, and education) and generational cohorts through the chi-square test (*χ*^2^). Then, three analyses of variance (ANOVA) were performed to assess associations of generational cohorts with: (1) gender identity milestones; (2) minority stress; and (3) mental health. In all ANOVAs, the generational cohort variable was included as fixed factor and gender identity milestones, minority stress, and mental health as dependent variables, respectively. The effect size was calculated using Cohen’s *f* (small effect = 0.10, medium effect = 0.25, and large effect = 0.40) [43]. Furthermore, we calculated between-group differences using Tukey’s post-hoc test. Finally, we also performed a series of independent sample *t*-tests to detect potential differences based on the sex assigned at birth (AFAB vs. AMAB) and gender identification (binary vs. nonbinary) on all variables resulted statistically associated with generational cohorts. In this case, the effect size was calculated using Cohen’s *d* (small effect = 0.20, medium effect = 0.50, and large effect = 0.80) [43].

## 3. Results

### 3.1. Relationships between Demographics and Generational Cohorts

As shown in Table 1, the only significant associations between demographics and generational cohorts were related to the sex assigned at birth and educational level. Specifically, the Generation Z cohorts were more likely to be in the trans-masculine spectrum than other gender groups, while Generation X cohorts were more likely to be in the trans-feminine spectrum than other groups. Furthermore, we found that the more the age cohort increased, the more AMAB participants rate was found. Additionally, the Generation Z cohorts showed higher educational levels than all other cohorts, and the more the age cohort increased, the more the educational level decreased. Instead, although percentages of binary gender identity were higher in the Generation X cohorts, contrary to Hypothesis 1, no statistically significant associations were found between generational cohort and type of gender identification (binary vs. nonbinary).

### 3.2. Gender Identity Milestones and Generational Cohorts

ANOVA revealed significant differences between the generational cohorts regarding self-labeling as TGNC and coming out as TGNC but not for first insights about being TGNC, partially confirming Hypothesis 2 (Table 2). Tukey post-hoc comparisons revealed several between-group differences showing that, although the mean age concerning the first insights to have a TGNC identity did not differ among groups, Generation X cohorts tended to self-label as TGNC and to come out as TGNC later than other cohorts. At the same time, Generation Z tended to self-label as TGNC and come out as TGNC earlier than other cohorts.

The independent sample *t*-test did not detect any difference on the mean age of gender identity milestones based on sex assigned at birth (all *p*s > 0.05). Instead, statistically significant differences emerged when gender identity was considered. Specifically, participants with a binary gender identity reported earlier ages for all the gender identity milestones than those with a nonbinary gender identity (Table 3).

### 3.3. Minority Stressors and Generational Cohorts

According to Hypothesis 3, ANOVA revealed that distal minority stressors did not differ among generational cohorts (Table 4). On the contrary, except for internalized transphobia, means of two proximal stressors (i.e., negative expectations and non-disclosure) significantly differed among groups. Specifically, Tukey post-hoc comparisons showed that the mean difference on negative expectations significantly differ only between Generation Z and Generation X cohorts (*MD* (mean difference) = 5.39, *SE* (standard error) = 1.71, *p* = 0.005), with younger participants having more negative expectations than older counterparts. Instead, Tukey post-hoc comparisons showed that the mean difference on non-disclosure significantly differed between both Generation Z and Millennials (*MD* = 2.37, *SE* = 0.81, *p* = 0.011) and Generation Z and Generation X (*MD* = 3.28, *SE* = 1.04, *p* = 0.005) but not between Millennials and Generation X (*MD* = 0.91, *SE* = 0.93, *p* = 0.593), indicating that younger participants had lower levels of disclosure than older counterparts.

The independent sample *t*-test did not detect any difference on negative expectations and non-disclosure based on sex assigned at birth (all *p*s > 0.05). Instead, except for non-disclosure, negative expectations differed based on gender identification (*t* = −4.07, *p* < 0.001, *d* = 0.69), with nonbinary participants (*M* = 20.94, *SD* = 6.93) showing higher negative expectations than binary individuals (*M* = 15.50, *SD* = 8.12).

### 3.4. Mental Health and Generational Cohorts

ANOVA revealed that means of all mental health measures significantly differed among groups (Table 5). Specifically, Tukey post-hoc comparisons showed that the mean difference on all negative mental health outcomes significantly differed between Generation Z and Generation X (anxiety: *MD* = 0.70, *SE* = 0.18, *p* < 0.001; depression: *MD* = 5.29, *SE* = 1.42, *p* = 0.001; general health: *MD* = 6.12, *SE* = 1.67, *p* = 0.001) and between Millennials and Generation X (anxiety: *MD* = 0.54, *SE* = 0.16, *p* = 0.002; depression: *MD* = 3.81, *SE* = 1.27, *p* = 0.008; general health: *MD* = 5.06, *SE* = 1.49, *p* = 0.002) but not between Generation Z and Millennials (all *p*s > 0.005), indicating that, according to the Hypothesis 4, younger participants had higher levels of mental health problems than older counterparts.

Again, the independent sample *t*-test did not detect any difference on mental health outcomes based on sex assigned at birth (all *p*s > 0.05). Instead, statistically significant differences emerged when gender identification was considered. Specifically, participants with a nonbinary gender identity reported greater severity than those with a binary identity on all mental health measures (Table 6).

## 4. Discussion

By integrating the MST and LCP, this study aimed at exploring potential differences among three generational cohorts of Italian TGNC individuals with respect to gender identity milestones, minority stress, and mental health. We found that, except for distal minority stressors, TGNC individuals have diverse experiences in terms of gender identity milestones, proximal minority stressors, and mental health across generational cohorts and gender groups. Overall, contrary to the sex assigned at birth, which was not associated with any of the studied outcome variables, our findings indicated the importance of considering both the generational differences and the gender identification in relation to minority stress and mental health of TGNC individuals.

Against the first hypothesis, although the percentage of nonbinary participants was higher in younger generational cohorts than older ones, we found that this difference was not statistically significant. This finding is not in line with previous studies, which found strong generational differences to gender identity [15,16,17]. It is plausible to hypothesize that the inconsistency of our result is due to the composition of the sample, as we could not include the Boomers group. Indeed, based on previous studies [15,16,17], it is very likely that older TGNC people would have identified themselves within a binary concept of gender and that this would have allowed to detect significant differences related to gender between younger and older participants. Thus, future Italian research should replicate this study including TGNC individuals who may be categorized as Boomers. However, it is also possible that nonbinary identities are more distributed across generational cohorts than is generally assumed by researchers. Indeed, it is plausible to hypothesize that older nonbinary cohorts use different language to describe their genders. Thus, future research should be thoughtful about the varied language that these nonbinary elders might use for their gender so as not to erase their experiences.

On the contrary, the finding concerning the greater likelihood of younger cohorts to self-identify in the trans-masculine spectrum (i.e., AFAB) compared to older cohorts is in line with recent studies, which have detected a temporal shift in the sex ratio among children and adolescents, with an impressive increase of AFAB youths being referred to specialized gender identity clinics [44,45]. However, it is hard to explain why this shift is happening, and the present research did not collect data that could help explain such generational difference.

Additionally, we found that younger generations were higher educated than their counterparts, as they had more access to university colleges. This finding may be interpreted as a positive effect of school anti-bullying policies, transgender visibility, and transgender rights advocacy that may prepare youths for post-secondary education by helping them to perceive university spaces as safer than in the past [25,46,47]. It may be the case in Italy, where the Italian Ministry of Education, Universities, and Research approved in 2015 a reform entitled *La Buona Scuola* (“The Good School”), that was aimed at introducing in all Italian school contexts educational measures to ensure equal opportunities for all students and to prevent gender- and sexual-based discrimination.

In support of our second hypothesis, we found that younger generations self-labeled and came out as TGNC earlier than older cohorts but that the mean age of first insights about being TGNC did not differ among cohorts. This finding reflects a generational shift in gender identity milestones that mainly concern the complete identification as TGNC (i.e., self-labeling and coming out). Such findings probably indicate that the socio-cultural context in which younger TGNC individuals live is perceived as more inclusive and accepting than that in which older generations lived, thus facilitating people to anticipate the realization of gender identity milestones [17]. For instance, a recent Italian study analyzed the experiences of parents of TGNC children and found that, despite difficulties in accessing information or benefitting from adequate social support, parents were personally committed in advocacy and affirmative actions, thus supporting their children and creating a safe family environment [48].

Instead, as first insights about being TGNC occur very early in life, often causing confusion, isolation, and shame [49], it is plausible to hypothesize that this milestone is less influenced by socio-cultural dynamics and that it mainly depends on internal psychological processes; this could explain why generational differences on this gender identity milestone were not detected. Interestingly, we found that participants with a nonbinary identity reported later ages for all gender identity milestones compared to those with a binary identity. In line with previous research [50], this finding may be explained by considering that binary and nonbinary transgender identity development follows diverse paths. Indeed, while the binary transgender identity development tends to follow a linear path, often resulting in a transition to a gender identity opposed to that assigned at birth (e.g., male-to-female or female-to-male), nonbinary transgender identity development is usually less linear and more flexible, and this can make the identification process more difficult and longer, deferring the time for the realization of all gender identity milestones [2,51].

Although younger TGNC individuals reported earlier ages for milestones, and this may be viewed as an effect of the positive changes in the social contexts in which they live [24], we also found that they reported more negative experiences on other study variables. Specifically, according to the third and fourth hypothesis, we found that, unlike distal minority stressors for which no generational differences were detected, younger participants had more negative expectations, lower levels of disclosure, and higher levels of mental health problems than the older counterparts. In line with Meyer et al. [24], it is plausible to hypothesize that no differences were detected about distal minority stressors (i.e., rejection, victimization, and discrimination) as, although the social environment has improved overall, it cannot be assumed that the same applies to all microenvironments; similarly, the improvement of the social environment does not automatically imply that the experiences of minority people have become free from developmental challenges.

For instance, in arguing the possible reasons why the most current studies are finding impressive rates of health disparities among sexual minority youths despite the positive social changes, Russell and Fish [52] talked about a “developmental collision” between normative adolescent developmental processes on the one hand and sexual minority identity development and visibility on the other. In other words, while the ages of identity milestones (in particular, the coming out) occur earlier than in the past, and this may be due to more inclusive environments than in the past, adolescence continues to be the developmental stage in which the adherence to sexual and gender norms represents an indicator of a cultural system regulating social relations more than other developmental stages, and this would increase the likelihood of being stigmatized if not adhering to such norms. This interpretative hypothesis could also explain why younger participants had higher negative expectations, lower levels of disclosure, and more significant mental health problems than older counterparts. To this end, within the MST, the perception of an environment as potentially stigmatizing would lead TGNC people to anticipate rejection and victimization, to not disclose their identity, and to develop mental health symptoms [3,53,54]. Furthermore, it is also plausible to hypothesize that older TGNC individuals have had more time than younger counterparts to integrate their TGNC identity into the self. Indeed, TGNC youths have had less time and existential possibilities than adults to learn adaptive coping strategies, and their responses to the minority stressors might be more maladaptive than those of their older counterparts [25].

Additionally, we also found that nonbinary people had higher negative expectations and mental health problems than binary individuals. This is in line with findings of previous studies that have highlighted that, compared with binary youths, nonbinary individuals receive lesser support from family and friends, participate less in activities taking place in their social environments, and are more likely to report negative mental health outcomes due to the invalidation of their identities [55,56,57].

Although this study fills a gap in the Italian scientific literature concerning TGNC people, significant limitations should be considered in interpreting the results. First, the study’s cross-sectional nature allows taking a picture of the sample in a specific moment of their life, not allowing to discuss historical changes and developmental trajectories in the status of TGNC people in society but only to theoretically interpret results as a manifestation of these changes. Future studies should implement longitudinal research designs to address this limitation. Second, we could not include in the sample the Boomers group due to its very small sample size. Third, as we conducted secondary analyses, information about two other important gender identity milestones (i.e., the age at which participants started to live full time as a TGNC person and underwent the first medical gender-affirmation procedure) are missing. Fourth, this study was conducted online, which prevented us from recruiting TGNC people who do not have access to the Internet. As suggested by Puckett et al. [17], the Internet is a crucial resource for TGNC people who need to reach information about transgender experiences, and this might mean that participants of the current study may be more likely to report gender identity milestones compared with other TGNC individuals who do not have access to the Internet. Lastly, almost all participants were Caucasian, which did not allow us to verify the role of ethnic diversity in the three main variables (i.e., gender identity milestones, minority stress, and mental health). Indeed, previous research has highlighted that TGNC individuals belonging to an ethnic minority experience additional stress related to racism and, therefore, are at greater risk of psychological distress [58]. Thus, future Italian studies should do their best to recruit more diversified samples of TGNC people in terms of ethnicity, also collecting more information on the ethnic status by asking, for example, if they were born in Italy or if they are migrants.

## 5. Conclusions

Despite limitations, the current study has some strengths. This is the first study that matched the MST and LCP in Italian TGNC people, highlighting the diversity of experiences related to gender identity milestones, minority stress, and mental health in different generational cohorts of Italian TGNC individuals. Thus, the study sheds light on the relationships between social contexts, identity development, stress, and health among Italian TGNC individuals. Overall, our findings have shown that, despite positive socio-cultural changes and visibility of TGNC populations, gender-based cultural systems that produce stigmatization processes towards gender minorities are still present in Italian society, as distal stressors did not differ among three generational cohorts analyzed, and such stressors are completely dependent on the social context. Thus, according to what was found by Meyer et al. [24], our findings seem to highlight that changes in the social contexts have a limited impact on stress processes and the health of TGNC individuals. This should lead Italian scholars, policymakers, and activists to continue to promote TGNC equality by implementing inclusive practices within the primary socialization environments in which TGNC people live (e.g., families, schools, and workplaces) and also by addressing equality messages to the whole society (e.g., through awareness campaigns or increasing the visibility of TGNC people in public contexts, such as television programs or political debates).

## Figures and Tables

**Table 1:** Demographics of the sample.

	Total Sample (18−54 Years) (*n* = 197)	Generation Z (18–22 Years) (*n* = 54)	Millennials (23–38 Years) (*n* = 106)	Generation X (39–54 Years) (*n* = 37)	
	*n* (%)	*n* (%)	*n* (%)	*n* (%)	*χ* ^2^
Sex assigned at birth					13.46 **
Male	63 (32)	12 (22.2)	30 (28.3)	21 (56.8)	
Female	134 (68)	42 (77.8)	76 (71.7)	16 (43.2)	
Gender identity					2.61
Binary	152 (77.2)	42 (77.8)	78 (73.6)	32 (86.5)	
Nonbinary	45 (22.8)	12 (22.2)	28 (26.4)	5 (13.5)	
Ethnicity					0.06
Caucasian	187 (94.9)	51 (94.4)	101 (95.3)	35 (94.6)	
Non-Caucasian	10 (5.1)	3 (5.6)	5 (4.7)	2 (5.4)	
Education					11.13 **
≤High school	100 (50.8)	18 (33.3)	57 (53.8)	25 (67.6)	
≥College	97 (49.2)	36 (66.7)	49 (46.2)	12 (32.4)	

** *p* < 0.01.

**Table 2:** Associations between gender identity milestones and generational cohorts.

	Age Cohorts								
	Generation Z (18–22 Years) (*n* = 54)		Millennials (23–38 Years) (*n* = 106)		Generation X (39–54 Years) (*n* = 37)				
	*M*	*SD*	*M*	*SD*	*M*	*SD*	*F*	*p*	*f*
Gender Identity Milestones									
First insight about being TGNC	10.49	5.37	9.72	6.45	11.03	7.85	0.63	0.532	0.08
Self-labeling as TGNC	17.39 ^a^	2.37	20.89 ^b^	6.52	25.86 ^c^	11.84	15.48	0.001	0.40
Coming out as TGNC	17.28 ^a^	2.85	21.83 ^b^	5.72	26.78 ^c^	13.94	17.83	0.001	0.43

*M*, mean; *SD*, standard deviation; *f*, Cohen’s *f*; TGNC, transgender and gender nonconforming. Means not sharing a superscript are significantly different from one another.

**Table 3:** Means comparisons of gender identity milestones based on gender identification.

	Gender Identification						
	Binary (*n* = 152)		Nonbinary (*n* = 45)				
	*M*	*SD*	*M*	*SD*	*t*	*p*	*d*
Gender Identity Milestones							
First insight about being TGNC	9.61	5.77	12.04	8.12	−2.24	0.026	0.34
Self-labeling as TGNC	20.25	7.58	22.82	7.32	−2.02	0.045	0.34
Coming out as TGNC	20.78	7.89	23.82	8.30	−2.24	0.026	0.37

*M*, mean; *SD*, standard deviation; *t*, Student’s *t*-test; *d*, Cohen’s *d*; TGNC, transgender and gender nonconforming.

**Table 4:** Associations between minority stress and generational cohorts.

	Age Cohorts								
	Generation Z (18–22 Years) (*n* = 54)		Millennials (23–38 Years) (*n* = 106)		Generation X (39–54 Years) (*n* = 37)				
	*M*	*SD*	*M*	*SD*	*M*	*SD*	*F*	*p*	*f*
Distal Minority Stressors									
Discrimination	1.96	1.24	2.18	1.42	2.13	1.46	0.44	0.641	0.07
Rejection	2.13	1.60	2.27	1.73	2.59	1.71	0.85	0.430	0.09
Victimization	1.56	1.30	2.32	1.85	1.65	1.87	2.39	0.070	0.11
Non-affirmation	13.18	7.40	11.74	7.76	10.29	7.87	1.58	0.209	0.13
Proximal Minority Stressors									
Internalized transphobia	13.07	8.15	12.17	8.79	10.51	9.47	0.95	0.390	0.10
Negative expectations	19.14 ^a^	7.66	16.57 ^ab^	7.84	13.75 ^b^	8.94	5.04	0.007	0.23
Non-disclosure	9.74 ^a^	4.72	7.37 ^bd^	4.91	6.46 ^cd^	5.01	6.08	0.003	0.25

*M*, mean; *SD,* standard deviation; *f*, Cohen’s *f*. Means sharing a superscript are not significantly different from one another.

**Table 5:** Associations between mental health and generational cohorts.

	Age Cohorts								
	Generation Z (18–22 Years) (*n* = 54)		Millennials (23–38 Years) (*n* = 106)		Generation X (39–54 Years) (*n* = 37)				
	*M*	*SD*	*M*	*SD*	*M*	*SD*	*F*	*p*	*f*
Mental health									
Anxiety	1.54 ^a^	0.82	1.39 ^ab^	0.90	0.84 ^c^	0.70	8.20	<0.001	0.29
Depression	12.29 ^a^	6.79	10.81 ^ab^	6.96	7.00 ^c^	5.36	7.22	0.001	0.27
General Health	17.79 ^a^	8.35	16.73 ^ab^	8.06	11.67 ^c^	5.89	7.57	0.001	0.28

*M*, mean; *SD*, standard deviation; *f*, Cohen’s *f.* Means sharing a superscript are not significantly different from one another.

**Table 6:** Means comparisons of mental health outcomes based on gender identification.

	Gender Identification						
	Binary (*n* = 152)	Nonbinary (*n* = 45)					
	*M*	*SD*	*M*	*SD*	*t*	*p*	*d*
Mental health							
Anxiety	1.23	0.87	1.67	0.79	−3.08	0.002	0.51
Depression	9.71	6.86	13.18	6.19	−3.04	0.003	0.52
General Health	15.46	8.01	18.22	7.88	−1.96	0.049	0.34

*M*, mean; *SD*, standard deviation; *t*, Student’s *t*-test; *d*, Cohen’s *d*.

## Data Availability

Anonymized data will be made available upon reasonable request to the corresponding author.
